# Stable Redox-Cycling Nitroxide Tempol Inhibits NET Formation

**DOI:** 10.3389/fimmu.2012.00391

**Published:** 2012-12-24

**Authors:** Ava Hosseinzadeh, Philipp K. Messer, Constantin F. Urban

**Affiliations:** ^1^Department of Molecular Biology, Umeå UniversityUmeå, Sweden; ^2^Umeå Centre for Microbial Research, Umeå UniversityUmeå, Sweden; ^3^Laboratory for Molecular Infection Medicine Sweden (MIMS), Umeå UniversityUmeå, Sweden; ^4^Department of Clinical Microbiology, Umeå UniversityUmeå, Sweden

**Keywords:** neutrophils, neutrophil extracellular traps, reactive oxygen species, nitroxide, Tempol, NET inhibition

## Abstract

To prevent the spread of pathogens neutrophils as the first line of defense are able to release Neutrophil Extracellular Traps (NETs), a recently discovered form of immune response. Reactive oxygen species (ROS) have been shown to be essential for many different induction routes of NET formation. Therefore, pharmacological inhibition of ROS generation has implications for research and medicine related to NETs. The application of diphenylene iodonium (DPI), an inhibitor of NADPH oxidase activity, is limited due to its toxicity to host cells as well as microbes. Therefore, we investigated the effect of 4-hydroxy-2,2,6,6-tetramethylpiperidine-1-oxyl (Tempol) a membrane-permeable radical scavenger on NET formation triggered by phorbol esters and *Candida albicans*. We quantified the amount of NETs with two complementary methods, using a microscopic analysis and an online fluorescence-based assay. In line with removal of ROS, Tempol reduced the amount of NET formation by neutrophils challenged with those stimuli significantly. Since Tempol efficiently blocks NET formation *in vitro*, it might be promising to test the effect of Tempol in experimental models of disorders in which NETs probably have hazardous effects.

## Introduction

Polymorphonuclear neutrophils (PMNs) are circulating leukocytes and serve as a first line of defense against microbial infections, but are at the same time a main contributor to hazardous effects during inflammation (Nathan, 2006). In humans, approximately 2 × 10^11^ neutrophils are produced per day (Borregaard, 2010). These professional phagocytes contain a large reservoir of antimicrobial proteins, which they secrete or release into the phagosome containing engulfed microbes (Amulic et al., 2012). The cells express the phagocyte NADPH oxidase complex which enables them to produce superoxide (Segal et al., 2012). The superoxide anion is converted spontaneously or enzymatically driven to other Reactive Oxygen Species (ROS). The exposure to antimicrobial proteins and ROS concertedly kills the phagocytosed microbe (Nathan, 2006).

Neutrophil Extracellular Traps (NETs) have been described as an extracellular mechanism of neutrophils to trap and kill microbes (Brinkmann et al., 2004). NETs consist of nuclear chromatin decorated with antimicrobial proteins. ROS are required to form NETs, as neutrophils from chronic granulomatous disease (CGD) patients, unable to form ROS, do not release NETs (Fuchs et al., 2007). NETs can be induced by various stimuli, such as bacteria, fungi, parasites chemokine IL-8, and protein kinase C activation via Phorbol Myristate Acetate (PMA; Brinkmann et al., 2004; Guimaraes-Costa et al., 2009; Urban et al., 2009; Marcos et al., 2010; Parker et al., 2012). As a consequence of NET release upon these stimuli the neutrophil plasma membrane ruptures and thus, per definition, the NET-releasing cell dies (Fuchs et al., 2007). Other studies proposed alternative mechanisms of DNA trap release that is catapult-like and involves the release of mitochondrial instead of nuclear DNA from viable neutrophils (Yousefi et al., 2009). *Staphylococcus aureus* seems to be able to induce NET formation by triggering the neutrophils to expel their nuclei packaged in vesicles leaving the plasma membrane intact (Pilsczek et al., 2010; Yipp et al., 2012). These findings illustrate the complexity of the process of NETosis and therefore the urgent need for better tools to study the underlying mechanisms in more detail.

Apart from their positive role during infection by preventing dissemination of microbes (Yipp et al., 2012), NETs are assumed to have hazardous effects as well. Thus, NETs may also contribute to harm the host during uncontrolled inflammatory processes. For instance, NETs have been described to be involved in autoimmune and inflammatory disorders, such as Small-Vessel Vasculitis (SVV; Kessenbrock et al., 2009; Nakazawa et al., 2012), Systemic Lupus Erythematosus (SLE; Hakkim et al., 2010b; Villanueva et al., 2011; Leffler et al., 2012), amyloidoses (Azevedo et al., 2012), and in Transfusion-induced Acute Lung Injury (TRALI; Caudrillier et al., 2012; Thomas et al., 2012). Treatment with vitamin C reduced the load of NETs during experimental TRALI in mice (Caudrillier et al., 2012). This in particular indicates that potential NET inhibitors could prove valuable in medical applications for diseases where NET formation is involved.

Since a significant proportion of described NET release mechanisms depend on ROS (Hakkim et al., 2010a), any molecule that prevents the generation of ROS or drives their metabolism should be able to inhibit the release of ROS-dependent NETs. Therefore, we reasoned that the ROS metabolizing compound Tempol (4-hydroxy-tetramethylpiperidin-1-oxyl) is able to interfere with NET formation. This low molecular weight compound is a stable, membrane-permeable redox-cycling nitroxide, mimetic to the super oxide dismutase (SOD) enzyme by scavenging superoxide radicals (Krishna et al., 1996). The great advantage of Tempol for any potential medical application is its very low toxicity in mammals (Wilcox, 2010). Other compounds that directly block ROS production, e.g., by inhibiting flavin enzymes of the NADPH oxidase complex or myeloperoxidase, such as diphenyleneiodonium (DPI) and sodium azide respectively, are either unspecific or toxic to cells (Riganti et al., 2004). Tempol can metabolize a variety of ROS and protect cells (Hahn et al., 1992) and animals (Goffman et al., 1992) from radiation damages.

To test the effect of Tempol on NET formation we used three different stimuli to induce NETosis: PMA, which has been shown to be ROS-dependent (Fuchs et al., 2007), the fungal pathogen *Candida albicans*, a physiological stimulus that also requires ROS (Ermert et al., 2009), and finally the chemokine IL-8, which has been suggested to induce NET formation ROS-independently (Marcos et al., 2010). For this purpose, the ROS production as well as the amount of netting neutrophils at different time points were determined microscopically and confirmed with an online fluorescence-based assay quantifying extracellular DNA or in more general terms cell death.

We show here that Tempol efficiently removes ROS produced by human neutrophils upon PMA and *C. albicans* stimulation, without negatively affecting phagocytic function of neutrophils. NET formation was inhibited by Tempol in a dose-dependent manner. PMA and *C. albicans*-induced NET formation efficiently as expected, whereas IL-8 did not trigger significant amounts of NETs. This study introduces Tempol as a valuable candidate compound to treat diseases in which NETs have hazardous effects to the host.

## Materials and Methods

### Isolation of neutrophils

Neutrophils were harvested from blood of healthy volunteers according to the recommendations of the local ethical committee (Regionala etikprövningsnämnden i Umeå) as approved in permit Dnr 09-210M. Fully informed consent was obtained, and all investigations were conducted according to the principles expressed in the Declaration of Helsinki. The isolation of neutrophils was performed as described previously (Fuchs et al., 2007). Briefly, neutrophils were separated from blood with two gradient centrifugation steps. The first one uses Histopaque 1119 (Sigma-Aldrich) to remove red blood cells while the second one uses discontinuous gradients of percoll (Amersham) that separate neutrophils from monocytes, lymphocytes, and residual red blood cells. Subsequently, the cells were washed with 1× PBS + 0.5% human serum albumin (HSA) and resuspended in RPMI 1640 medium without phenol red (Lonza).

### Culture of *C. albicans*

An overnight culture of *C. albicans* (SC5314) was inoculated in YPD from a culture dish. After incubation the culture was diluted to a starting OD_600_ of 0.1 in fresh growth medium. To grow *C. albicans* in its yeast form the cells were incubated in YPD at 30°C for 4 h. To produce hyphae the same dilution was used in RPMI medium and the cell suspensions were incubated at 37°C for 4 h. The multiplicity of infection (MOI) for *C. albicans* hyphae was adjusted to the initial number of yeast cells that were used for inoculation, since under these conditions almost 100% filamentation can be expected. The factor 3 × 10^7^ cells/ml at OD_600_ of 1 was used to convert the number of yeast cells in the logarithmic phase to the number of hyphae.

### Luminol assay

Neutrophil ROS production was determined by luminol bioluminescence as described previously (Ermert et al., 2009). Briefly, cells were seeded in a white 96 well plate at a concentration of 5 × 10^4^ cells per well. Twenty microliters of Tempol in different concentrations or RPMI were added and the cells incubated for 15 min at 37°C. For the assay, luminol (Sigma-Aldrich) and horseradish peroxidase (Sigma-Aldrich) were added to a final concentration of 50 μM and 1.2 U/ml respectively. NET formation was induced just before starting the assay by the addition of PMA at final concentration of 100 nM, yeast or hyphae of *C. albicans* at an MOI of 3, or human recombinant IL-8 at a final concentration of 100 nM (Abcam). ROS were measured by luminescence catalysis in a Tecan Infinite 200 plate reader every 3 min for a period of 3 h.

### Cell death assay

Neutrophil cell death or presence of extracellular DNA was measured by fluorescence with Sytox Green (Invitrogen) similar to previous descriptions (Fuchs et al., 2007; Ermert et al., 2009). Briefly, cells were seeded in a black 96 well plate with a concentration of 5 × 10^4^ cells per well in a total volume of 160 μl. Subsequently, Tempol was added in different concentrations and plates were incubated for 15 min at 37°C. After this short incubation, Sytox Green, a membrane-impermeable DNA dye, was added to a final concentration of 2.5 μM, before cells were stimulated with PMA (100 nM final concentration), *C. albicans* yeast or hyphae (both MOI 3) or IL-8 (100 nM). The samples were monitored for fluorescence under cell culture conditions (37°C, 5% CO_2_) in a plate-based fluorescence spectrophotometer (Fluostar Omega, BMG) in intervals of 10 min for a total of 10 h. Percent NET formation was calculated as percentage of lysis control. From this value, percent cell death of unstimulated neutrophils was subtracted at the respective time points. Data were presented as percent cell death.

### Phagocytosis assay

Neutrophil phagocytosis was measured by using pHrodo *S. aureus* bioparticles according to the manufacturer’s protocol (Invitrogen). This assay is based on increased fluorescence of the beads upon acidification in the phagosome.

Briefly, cells were seeded in a white 96 well plate at a concentration of 5 × 10^4^ cells per well and pHrodo *S. aureus* BioParticles were added at a final concentration of 25 μg/well in a total volume of 150 μl. Samples containing Cytochalasin D (Sigma-Aldrich) at a final concentration of 10 μg/ml and incubated for 15 min at 37°C to block phagocytosis served as a negative control. As 100% control BioParticles in buffer at pH 4 were used. Tempol or RPMI was added at different concentrations. Plates were measured in a fluorescence spectrophotometer (Fluostar Omega, BMG) every 30 min for 2 h.

### Chemotaxis assay for human neutrophils using transwell inlets

To test bioactivity of human recombinant IL-8 (Abcam) neutrophil migration was measured in a transwell system. The neutrophils were labeled with a fluorescent cytoplasmic dye, BCECF-AM (Sigma-Aldrich) at a final concentration of 3.3 μM and 5 × 10^5^ neutrophils were seeded on a fluorescence-impermeable transwell membrane (BD Falcon, HTS FluoroBlok Insert, 3.0 μM pore size). The transwell was placed into a 24-well tissue culture plate (BD Falcon) just before measurement. The lower compartment of the tissue culture plate contained 600 μl of RPMI with 0.05% HSA and 10 nM human recombinant IL-8 was added as chemoattractant. For base line migration the lower compartment contained medium only. As 100% control served 5 × 10^5^ BCECF-AM labeled neutrophils without inserting the transwell system. The samples were monitored for fluorescence under cell culture conditions (37°C, 5% CO_2_) in a fluorescence spectrophotometer (Fluostar Omega, BMG) in intervals of 1 min for a total of 10 min. Chemotaxis was plotted as percentage of total signal and base line signal was subtracted.

### Immunostaining of neutrophils

Cells were seeded on cover slips coated with 0.01% poly-l-lysine (Sigma-Aldrich) in 24-well plates with a concentration of 1 × 10^5^ cells per well in a total volume of 500 μl. Tempol was added to a final concentration of 30 mM and incubated for 15 min. Subsequently, cells were induced with PMA (100 nM final concentration) *C. albicans* yeast or hyphae (MOI 3) or IL-8 (100 nM) for 3 and 6 h at 37°C and 5% CO_2_. After incubation the cells were fixed using paraformaldehyde (2% final concentration) for 30 min at RT. Specimens were stored at 4°C. For immune staining the cover slips were washed three times with PBS and permeabilized with 0.5% TritonX-100 (Merck) in PBS. Cells were blocked at 37°C for 1 h in blocking buffer containing 3% cold water fish gelatin (Sigma-Aldrich), 1% gelatin from bovine serum (Sigma-Aldrich), 5% donkey serum (Jackson Immuno research), and 0.25% Tween 20 (VWR). Antibodies directed against histone H1 (1.25 μg/ml; #BM465, Acris) and directed against neutrophil elastase (6 μg/ml; #481001, Calbiochem) were used and incubated for 1 h at 37°C. Secondary antibodies conjugated to Cyanine dyes (Cy 2 and Cy 3, Jackson Immuno research) were used. DNA was stained with DAPI and washed one time with double distilled water. Specimens were mounted in Mowiol 4-88 (Calbiochem) and images were captured using a 20× objective (Nikon 90i fluorescence microscope). The neutrophil elastase staining was confirmatory to validate the presence of NETs and is not presented here. Images were analyzed with NIS-Elements analysis software ver. 3.20.

### Microscopic quantification of NETs

We adapted previously described methods for NET quantification microscopically, such as Ermert et al. (2009), Papayannopoulos et al. (2010), Keshari et al. (2012), Parker et al. (2012). Microscopic image analysis was performed using histone immune-stained samples including five random images and counting at least 400 cells from each condition. ImageJ version 1.44p software was used to measure pixel areas by adjusting the threshold above background. Measured pixel areas were converted to μm^2^ (With 200-fold magnification 472 pixels equal 100 μm^2^). Particles comprising an area of less than 15 μm^2^ were excluded from analysis. Human neutrophils have an average diameter of 10 μm, hence their average area in an unstimulated stage is approximately 80 μm^2^ (assuming a circle and using π × *r*^2^). To quantify the number of cells that underwent NET formation we chose signal events that exceeded 100 μm^2^ and thus were larger than the whole intact cell area. Events larger than 100 μm^2^ were either considered as decondensed nuclei, an essential step prior to NETosis, or as released NETs. The total number of cells was counted and NET-forming cells were expressed as percentages of events larger than 100 μm^2^ from the total number of events. Three independent experiments using neutrophils from three different donors were analyzed for each sample.

### Statistical analysis

Tukey one-way ANOVA was applied for statistical analysis of the data using the software GraphPad Prism v 5.00.

## Results

### Tempol scavenges ROS produced by PMA-stimulated neutrophils

**Figure 1:**
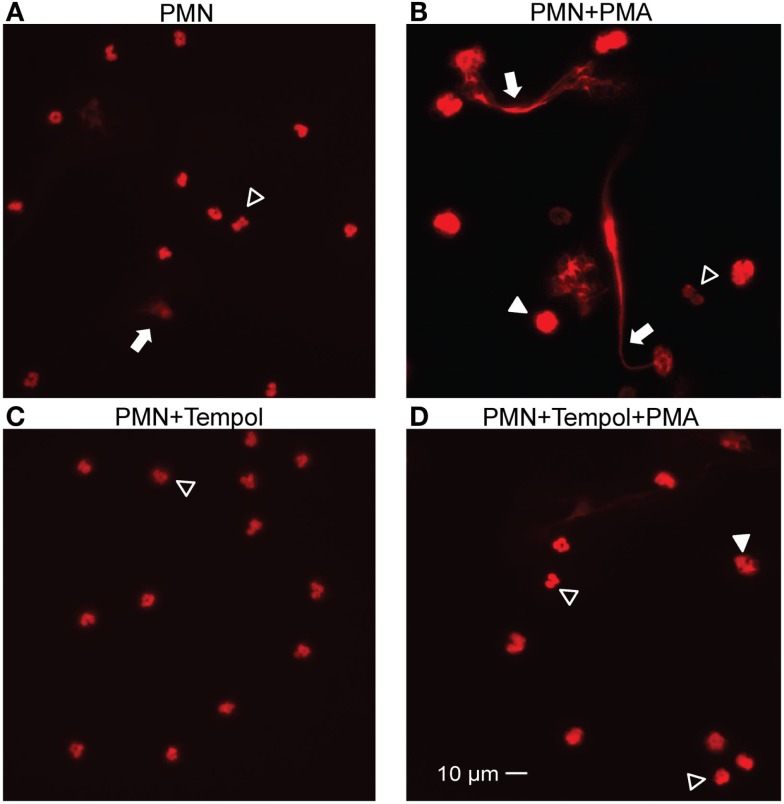
**Tempol reduces NET formation stimulated by PMA**. Indirect immunofluorescence of PMA-stimulated PMNs (100 nM) for 6 h in the presence or absence of Tempol (30 mM). Unstimulated PMNs and PMNs incubated with Tempol only served as control. NET formation is visualized after fixation using staining of chromatin with a primary antibody directed against histone H1 and a Cy-3 conjugated secondary antibody. The representative microscopic images illustrate unstimulated PMNs **(A)**, PMNs stimulated with PMA **(B)**, PMNs treated with Tempol **(C)**, and PMNs treated with Tempol and stimulated with PMA **(D)**. NETs are indicated by arrows, decondensed nuclei with filled arrow heads. Open arrow heads show intact PMNs. Images were captured with a Nikon Eclipse 90i microscope and a Hamamatsu Orca-ER charge-coupled device camera using a 40× objective. Scale bar: 10 μm.

Protein kinase C (PKC) stimulation by the phorbol ester PMA is a well-known strong inducer of NET formation (Brinkmann et al., 2004). PMA-induced NET formation has additionally been shown to be ROS-dependent (Fuchs et al., 2007). Therefore, we tested the ROS scavenger Tempol for inhibition of NET formation. We stimulated neutrophils with PMA that have either been previously incubated with Tempol or with culture medium only. We analyzed the samples by microscopic immunofluorescence as described in Materials and Methods. After 6 h of incubation we observed NET formation in the mock-treated specimen. Patchy, web-like structures were present that stained positive for histone, a major component of NETs, and decondensation of nuclear chromatin could be observed (Figure 1). Decondensation of neutrophil nuclei is a hallmark of NET formation and occurs previous to the release of NETs (Fuchs et al., 2007). In contrast, the Tempol-treated neutrophils formed significantly less NETs and the majority of nuclei were lobulated indicating that they were still intact (Figure 1). Thus, we concluded that Tempol has NET-inhibitory activity.

**Figure 2:**
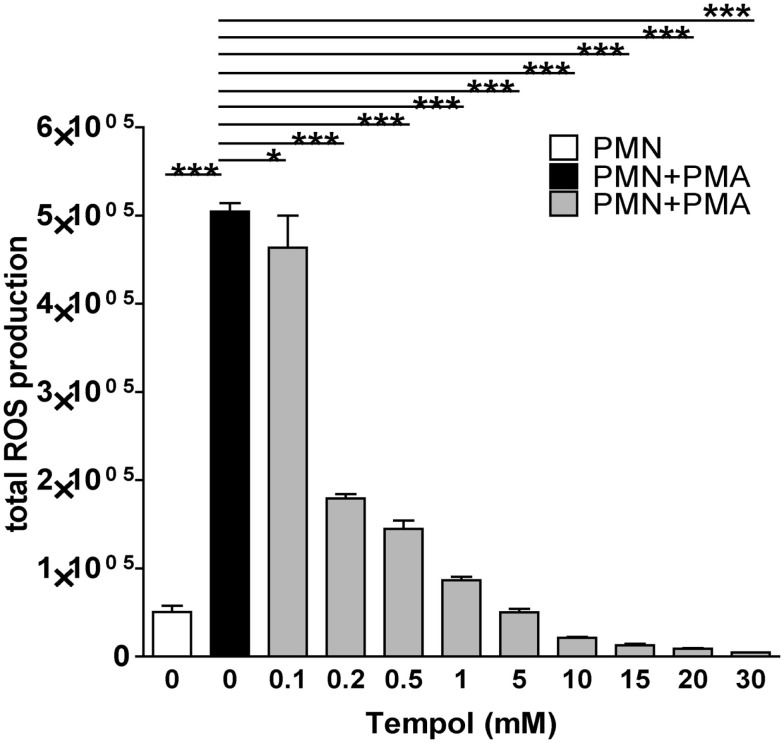
**Tempol scavenges neutrophil ROS triggered by PMA**. The total amount of ROS generated by PMNs over the course of three 3 h is plotted against increasing concentrations of Tempol. The half maximal inhibitory concentration (IC50) is 0.2 mM Tempol. ROS amounts were calculated as area under the curve (AUC). White bar: unstimulated PMNs, black bar: PMNs stimulated with 100 nM PMA, grey bars: PMNs treated with different concentration of Tempol prior to stimulation with PMA. Significance was analyzed by Tukey one-way ANOVA (*P ≤ 0.05 and ***P ≤ 0.001). One representative experiment out of three independent experiments with three different donors is shown. Data are presented as means of three technical replicates ±SD.

First, we aimed to identify adequate concentrations of Tempol to remove ROS produced by neutrophils. For this purpose, we induced neutrophils with PMA in the presence of different concentrations of Tempol and subsequently measured the amount of ROS produced in a luminol-based plate assay. Tempol at a concentration of 0.2 mM removed approximately 50%, 1 mM 75% and 10 mM more than 90% of PMA-induced ROS (Figure 2). Thus, Tempol was able to remove PMA-induced neutrophil ROS efficiently and in a dose-dependent manner.

### Tempol inhibits PMA-induced ROS-dependent NET formation

**Figure 3:**
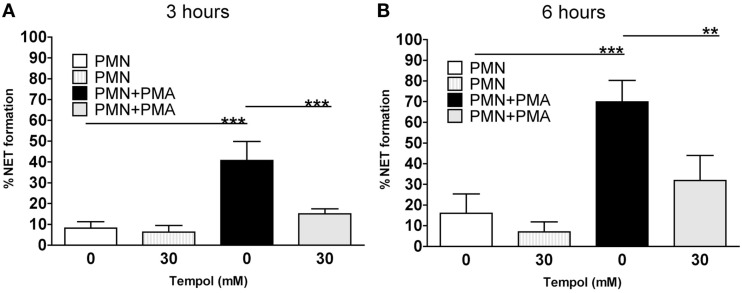
**Tempol prevents PMA-induced NET formation**. Microscopic evaluation of NET formation by PMNs stimulated with 100 nM PMA reveals reduced formation of NETs in the presence of Tempol. Percentage of NET formation after 3 h **(A)** and after 6 h **(B)**. White bars: unstimulated PMNs, dashed gray bars: PMNs treated with Tempol only (30 mM), black bars: PMNs stimulated with PMA, gray bars: Tempol-treated PMNs stimulated with PMA. The percentage of cells undergoing NET formation was computed by using ImageJ as explained in materials and methods. Significance was analyzed by Tukey one-way ANOVA (**P ≤ 0.01 and ***P ≤ 0.001). Data are presented as means ± SD of three independent experiments with three different donors.

We next investigated the effect of Tempol on NET formation in a quantitative manner. To unambiguously determine NET formation we chose a microscopic approach. Neutrophils were stimulated with PMA in the presence of 0, 15, or 30 mM Tempol and then fixed after 3 and 6 h, respectively. The cells were labeled with an anti-histone antibody and analyzed by immunofluorescence microscopy. We adapted previously described methods to quantify NET formation microscopically, such as Ermert et al. (2009), Papayannopoulos et al. (2010), Keshari et al. (2012), Parker et al. (2012). Determining the area occupied by intact cells or NETs – as expressed in μm^2^ – respectively, provides an appropriate measure to distinguish NETs from intact or non-NETotic dead neutrophils and thereby representing a means to assay NET formation in a quantitative manner (see Materials and Methods). Using this microscopic approach we determined that 40% of PMA-stimulated neutrophils underwent NETosis after 3 h and 70% after 6 h (Figure 3). Notably, 30 mM Tempol reduced NET formation significantly to 15% after 3 h and to 30% after 6 h (Figure 3). Background levels of NETs from unstimulated neutrophils were usually around 20% after 6 h of incubation. In conclusion, Tempol treatment reduced PMA-induced NET formation to background levels, comparable to unstimulated neutrophils (Figure 3B).

To confirm these results with a microscopy-independent assay, we used a fluorescence-based cell death assay (CDA) taking advantage of the fact that the plasma membrane ruptures during NET release (Ermert et al., 2009). Neutrophils were treated with PMA in the presence of the membrane-impermeable DNA dye Sytox Green, only detecting extracellular DNA or DNA that is not surrounded by an intact membrane. The samples were monitored for fluorescence under cell culture conditions. NET formation was calculated as percentage of lysis control (equaling 100%) and additionally, background levels from unstimulated neutrophils were subtracted (see Materials and Methods).

**Figure 4:**
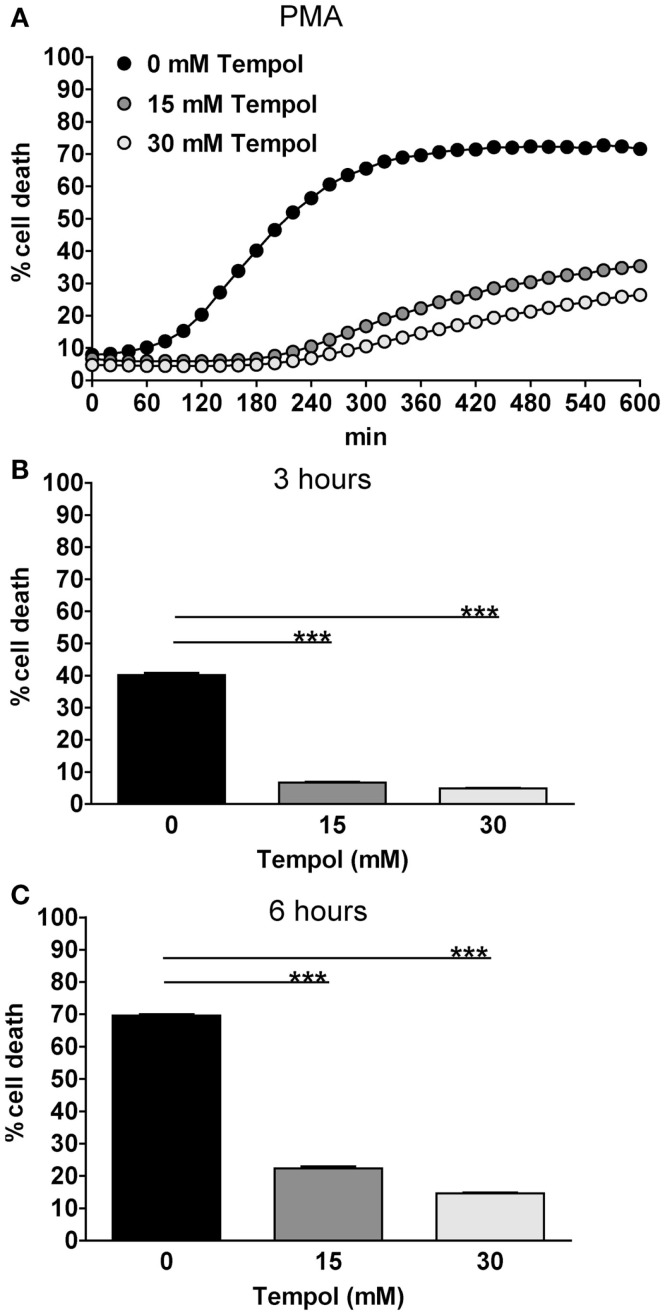
**Tempol prevents PMA-induced NET formation quantified by cell death assay**. Tempol reduces PMN cell death in a dose-dependent manner. The rate of cell death of PMNs stimulated with the highly potent NET-inducer PMA in the presence or absence of Tempol was analyzed over a period of 10 h by measurement of DNA-staining by the cell-impermeable dye Sytox Green. **(A)** The *Y*-axis represents the relative amount of dead cells after normalization to the lysis control (100%). Background values (unstimulated cells) were subtracted from all values. To allow better comparison with microscopic analyses, the effect of Tempol on PMA-induced PMN cell death after stimulation for 3 h **(B)** and 6 h **(C)** is additionally depicted as bar panels. **(A)** Black curve: PMNs stimulated with 100 nM PMA without Tempol treatment, gray curves: PMNs treated with different concentrations of Tempol prior to stimulation with PMA. Significance was analyzed by Tukey one-way ANOVA ***P ≤ 0.001). One representative experiment out of three independent experiments with three different donors is shown. Data are presented as means of three technical replicates ±SD.

**Figure A1:**
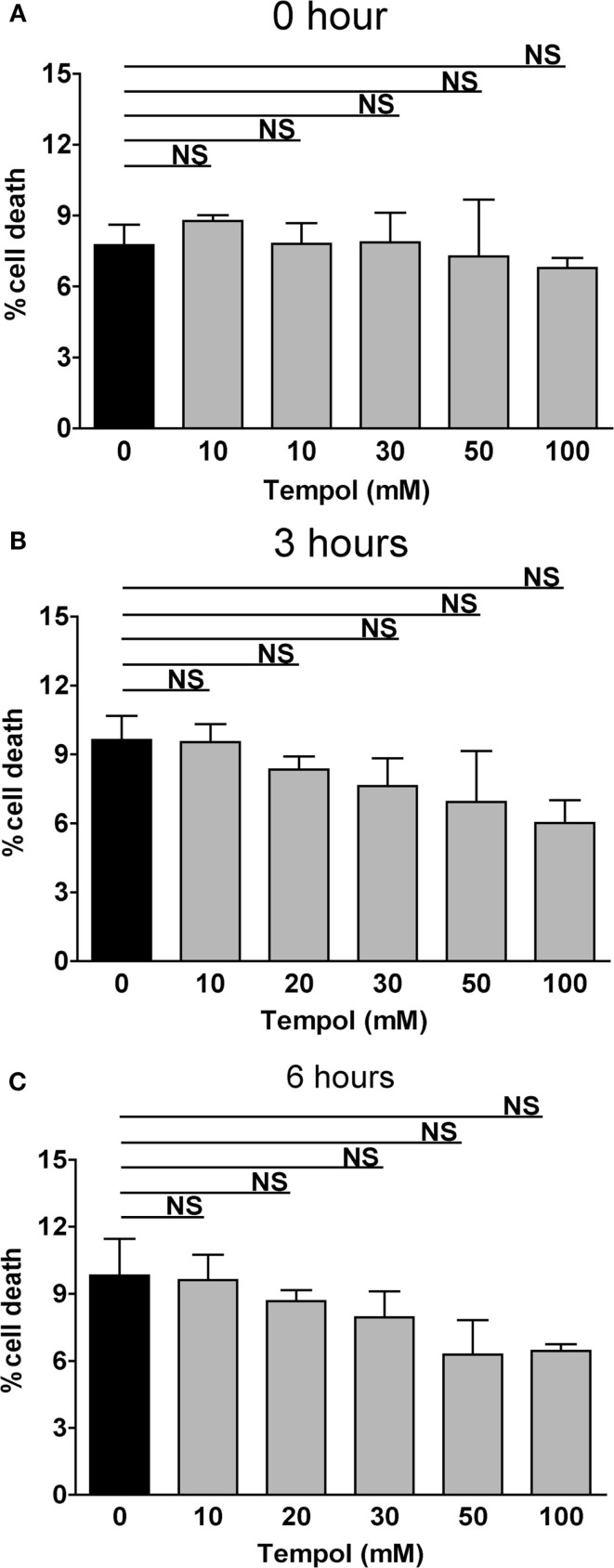
**Tempol alone is not toxic to human neutrophils**. The rate of cell death of PMNs stimulated with Tempol was analyzed over a period of 6 h by measurement of DNA-staining by the cell-impermeable dye Sytox Green. The *Y*-axis represents the relative amount of dead cells after normalization to the lysis control (100%). PMNs treated with Tempol in a dose-dependent manner at time point 0 **(A)**, after 3 h **(B)**, and after 6 h **(C)**. Black bars: unstimulated PMNs, gray bars: PMNs treated with Tempol at different concentrations. Significance was analyzed by Tukey one-way ANOVA (NS: *P* > 0.05). One representative experiment out of three independent experiments with three different donors is shown. Data are presented as means of three technical replicates ±SD.

In accordance to the microscopic analysis, Tempol reduced PMA-induced NET formation, as indicated by a decrease in fluorescence in the corresponding samples (Figure 4A). To be able to directly compare these results to the microscopic analysis, we extracted the values at 3 and 6 h from these measurements, respectively. The effect of Tempol was dose-dependent resulting in less than 10% cell death after 3 h (Figure 4B) and 20% cell death at 15 mM as well as 15% cell death at 30 mM Tempol after 6 h (Figure 4C). A specific mode of action for Tempol in reducing NET release is therefore highly probable. Notably, Tempol even decreased spontaneous cell death in the unstimulated control as verified by the same assay up to concentrations of 100 mM, clearly demonstrating that Tempol has very low toxicity at these concentrations (Figure A1 in Appendix).

Taken together both the microscopic analysis and CDA confirmed that Tempol is able to block NET formation efficiently. Moreover, both assays resulted in highly similar levels of NET formation by PMA-stimulated neutrophils, suggesting that both assays are suitable to quantify NET formation.

### Tempol scavenges ROS produced by *C. albicans*-stimulated neutrophils

Since PMA is a very potent, however non-physiological stimulus of NET formation, we used *C. albicans* to induce NETosis in neutrophils representing the most frequent fungal pathogen in humans. *C. albicans* is dimorphic (Sudbery et al., 2004) and both growth forms, yeast cells as well as filamentous hyphae are able to trigger NET formation by neutrophils (Urban et al., 2006). Therefore, we included both growth morphologies in this study.

**Figure 5:**
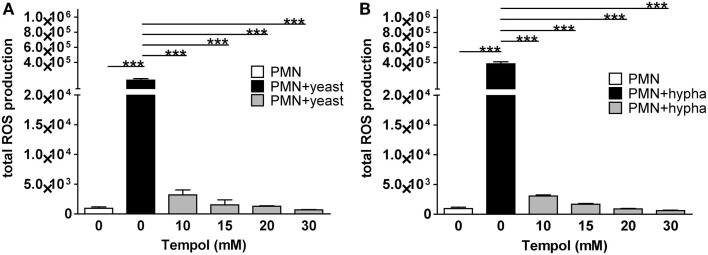
**Tempol scavenges neutrophil ROS triggered by different growth forms of *C. albicans***. The total amount of ROS generated by PMNs is plotted against increasing concentrations of Tempol present in the culture medium. PMNs were infected with *C. albicans* (MOI 3) in the presence of different concentrations of Tempol. The total amount of ROS produced over 3 h was calculated as area under the curve (AUC). PMNs infected with of *C. albicans* yeast **(A)** and hyphal cells **(B)**, respectively. White bars: unstimulated PMN, black bars: PMNs infected with *C. albicans*, gray bars: PMNs treated with different concentration of Tempol prior to stimulation with fungi. Significance was analyzed by Tukey one-way ANOVA (***P ≤ 0.001). One representative experiment out of three independent experiments with three different donors is shown. Data are presented as means of three technical replicates ±SD.

To identify the Tempol concentrations which are sufficient to remove neutrophil ROS triggered by *C. albicans* and consequently potentially abolish NET formation, we measured ROS production in the presence of 0 mM up to 30 mM Tempol in the infection assays (Figure 5). Ten millimolar Tempol reduced yeast-triggered neutrophil ROS down to 2.5% of the amount produced in the absence of Tempol. ROS were further reduced in a dose-dependent manner to 0.5% at 30 mM Tempol. Similarly, hyphae-triggered ROS was reduced by Tempol, but with no further decrease at 30 mM Tempol. We concluded that 30 mM Tempol is sufficient to efficiently remove *C. albicans*-induced neutrophil ROS.

### Tempol inhibits *C. albicans*-induced ROS-dependent NET formation

**Figure 6:**
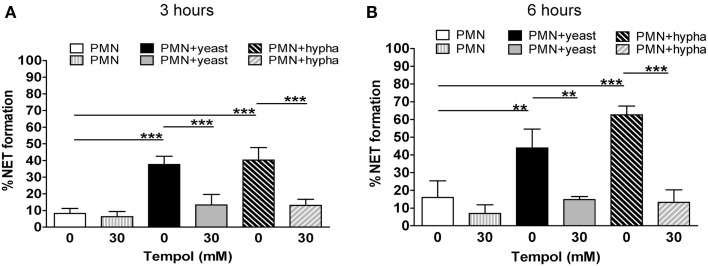
**Tempol prevents NET formation induced by different growth forms of *C. albicans***. Microscopic evaluation of *C. albicans* infected PMNs (MOI 3) demonstrates reduction of NET formation by Tempol. Percentage of NET formation after infection with *C. albicans* yeast or hyphal cells for 3 h **(A)** and 6 h **(B)**. White bars: unstimulated PMNs, dashed gray bars: PMNs treated with Tempol, black bars: PMNs stimulated with *C. albicans* yeast growth form, gray bars: PMNs treated with Tempol prior to infection with yeast, diagonal dashed black bars: PMNs stimulated with *C. albicans* hyphae, diagonal dashed gray bars: PMNs treated with Tempol prior to infection with hyphae. The percentage of cells undergoing NET formation was calculated as explained in materials and methods. Significance was analyzed by Tukey one-way ANOVA (**P ≤ 0.01 and ***P ≤ 0.001). Data are presented as means ±SD of three independent experiments with three different donors.

As *C. albicans* induces NET formation in a strictly ROS-dependent manner (Ermert et al., 2009), we next investigated the effect of Tempol on NET formation induced by the fungal pathogen using the microscopic analysis described above. In these assays, yeasts induced 35% NETs after 3 h and 45% after 6 h of incubation (Figures 6A,B; solid bars). Thirty millimolar Tempol significantly reduced NET formation at 3 h to approximately 10% and at 6 h to 15% after stimulation with *C. albicans* yeasts. Similar results were obtained when hyphae were used to trigger NET formation (Figures 6A,B; dashed bars). Tempol reduced NET formation from 40% after 3 h and 65% after 6 h to 10 and 15%, respectively. Consequently, 30 mM Tempol was able to reduce NET formation induced by *C. albicans* yeast and hyphae to background levels.

**Figure 7:**
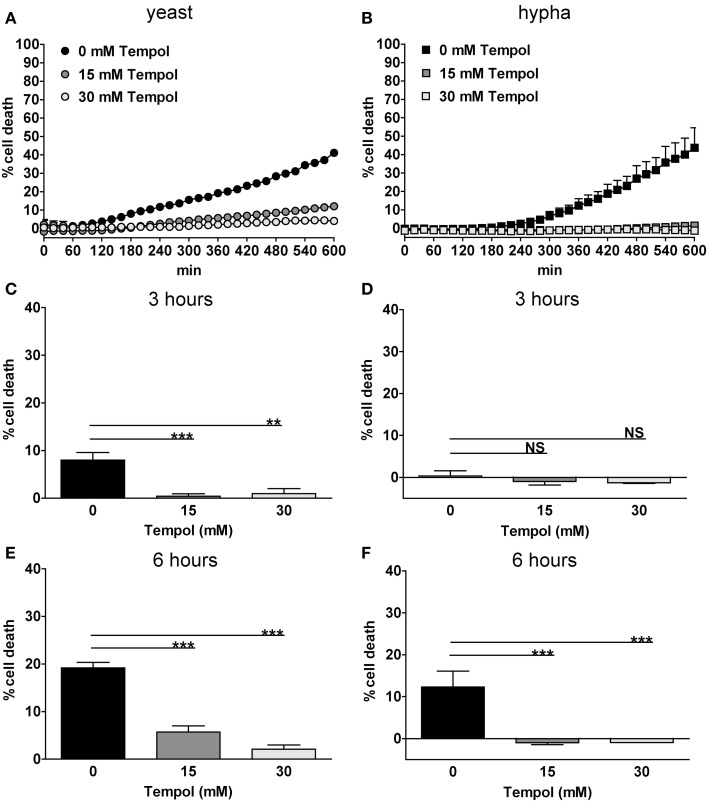
**Tempol prevents *C. albicans*-induced NET formation quantified by cell death assay**. Tempol reduces cell death during infection with *C. albicans*. PMN cell death rates after infection with different growth morphologies of *C. albicans* in the presence or absence of Tempol were analyzed over a period of 10 h by measurement of DNA-staining by the cell-impermeable dye Sytox Green. The *Y*-axis represents the relative amount of dead cells after normalization to the lysis control (100%). Background values (unstimulated cells) were subtracted from all values. PMN cell death induced by the yeast **(A)** or the hyphal **(B)** growth form of *C. albicans* (MOI 3). Black curves: PMNs infected with *C. albicans*, gray curves: PMNs infected with *C. albicans* in the presence of different concentrations of Tempol. To allow for better comparison with microscopic analyses, the effect of Tempol on PMN cell death after infection with *C. albicans* at 3 h **(C,D)** and 6 h **(E,F)** time points was compared in respect of the different cell morphotypes used. Significance was analyzed by Tukey one-way ANOVA (NS: *P* > 0.05, **P ≤ 0.01, and ***P ≤ 0.001). One representative experiment out of three independent experiments with three different donors is shown. Data are presented as means of three technical replicates ±SD.

To confirm the microscopic quantification we used the CDA (Figure 7) as described for PMA-stimulated neutrophils. Upon infection with yeast *C. albicans*, 40% neutrophil death was observed (Figure 7A) and 45% neutrophil death upon hyphae (Figure 7B) after 10 h of infection. To directly compare these results to the microscopic analysis, we extracted the values at 3 and 6 h, respectively (Figures 7C–F). Yeast induced 10 and 20% cell death above background of unstimulated neutrophils after 3 and 6 h, respectively (Figures 7C,E). Tempol at 15 and 30 mM reduced neutrophil cell death below 10% at all time points (Figures 7C,E). When hyphae were used for infection we observed 10% increase of neutrophil cell death above background after 6 h (Figures 7D,F). Again, Tempol reduced neutrophil cell death triggered by hyphae to 0%, indicating that Tempol inhibits ROS-dependent NET formation.

Taken together, the results from the microscopic quantification and from the CDA are confirmatory and thus advantageous to singular assays. As the CDA is much less laborious as the microscopic analysis, this assay represents a valuable tool to quantify NET formation in a simple and robust manner.

### IL-8 and *C. albicans* co-stimulation does not increase NET formation by human neutrophils

**Figure 8:**
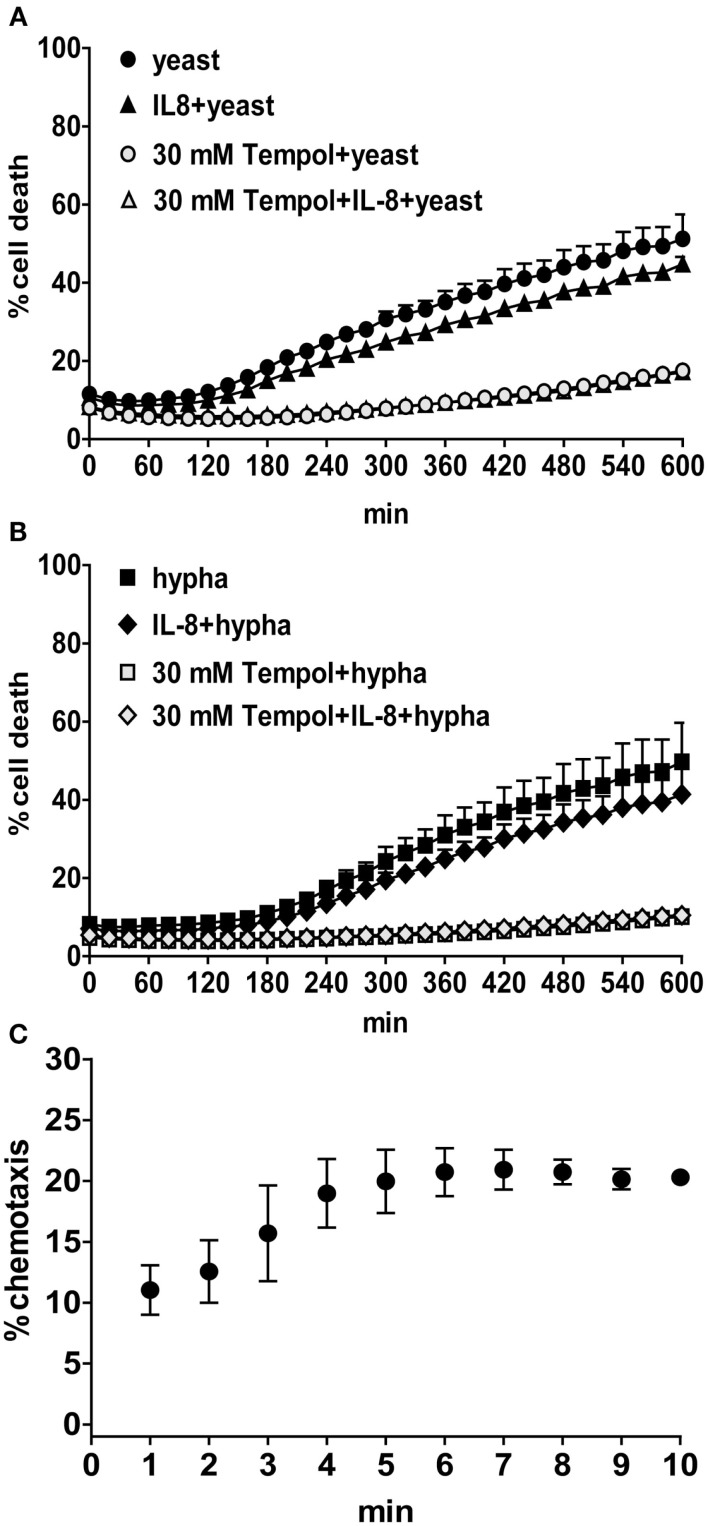
**During infection with *C. albicans* IL-8 does not enhance neutrophil NET formation**. The rate of cell death of PMNs treated with 100 nM IL-8 for 30 min prior to infection with *C. albicans* (MOI 3) was analyzed over a period of 10 h by measurement of DNA-staining by the cell-impermeable dye Sytox Green. The *Y*-axis represents the relative amount of dead cells after normalization to the lysis control (100%). Background values (unstimulated cells) were subtracted from all values. Black symbols: PMNs infected with yeast [**(A)**, circles and triangles] or hyphal cells [**(B)**, squares and rhombuses] and additionally with IL-8 (triangles and rhombuses); gray symbols: PMNs additionally treated with 30 mM Tempol. One representative experiment out of three independent experiments with three different donors is shown. Data are presented as means of three technical replicates ±SD. Neutrophil chemotaxis was measured in a transwell assay **(C)**. The *Y*-axis represent the percentage of neutrophil migration toward 10 nM IL-8 normalized to 100% florescent labeled cells. The base line levels (migrated cells without chemoattractant) were subtracted from all values. One representative experiment out of three independent experiments with three different donors is shown. Data are presented as means of three technical replicates ±SD.

Since IL-8 was shown to prime neutrophils to produce more ROS upon stimulation with the staphylococcal product fMLP (Brechard et al., 2005), we aimed to elucidate whether treatment of neutrophils with IL-8 prior to infection with both morphotypes of *C. albicans* additionally enhances neutrophil NET formation. However, IL-8 and *C. albicans* did not synergize to result in increased NET formation as observed using the CDA (Figures 8A,B). The bioactivity of recombinant IL-8 was confirmed with a neutrophil chemotaxis assay showing that human neutrophils migrated significantly toward the chemoattractant (Figure 8C). We also investigated the contribution of IL-8 alone to ROS production and NET formation. We stimulated neutrophils with 100 nM IL-8 as described previously (Marcos et al., 2010). Compared to PMA or *C. albicans*, in our hands IL-8 did not induce significant amounts of ROS and neither a significant increase in NET formation as shown by microscopic analysis and the CDA (data not shown).

## Discussion

**Figure A2:**
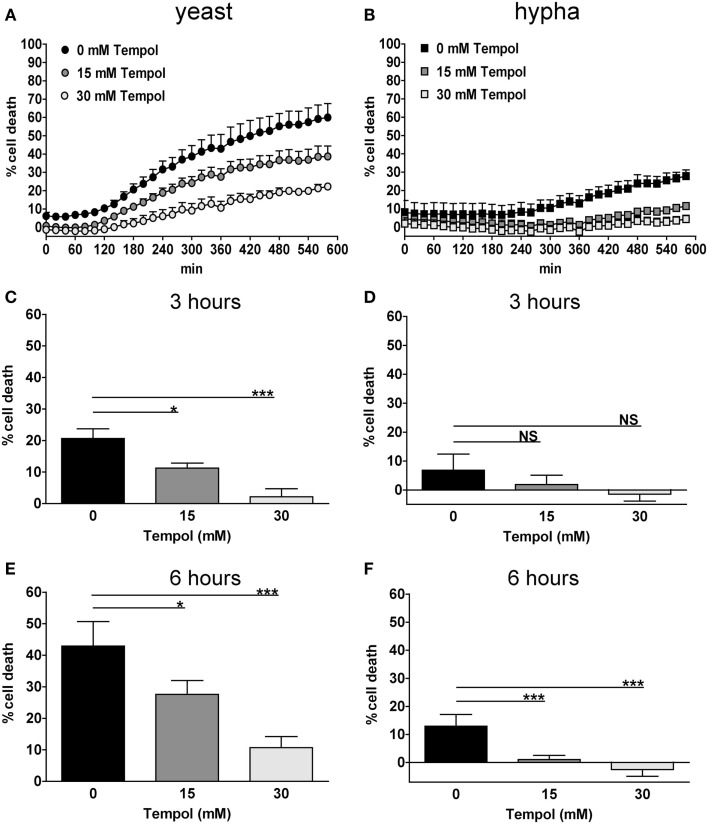

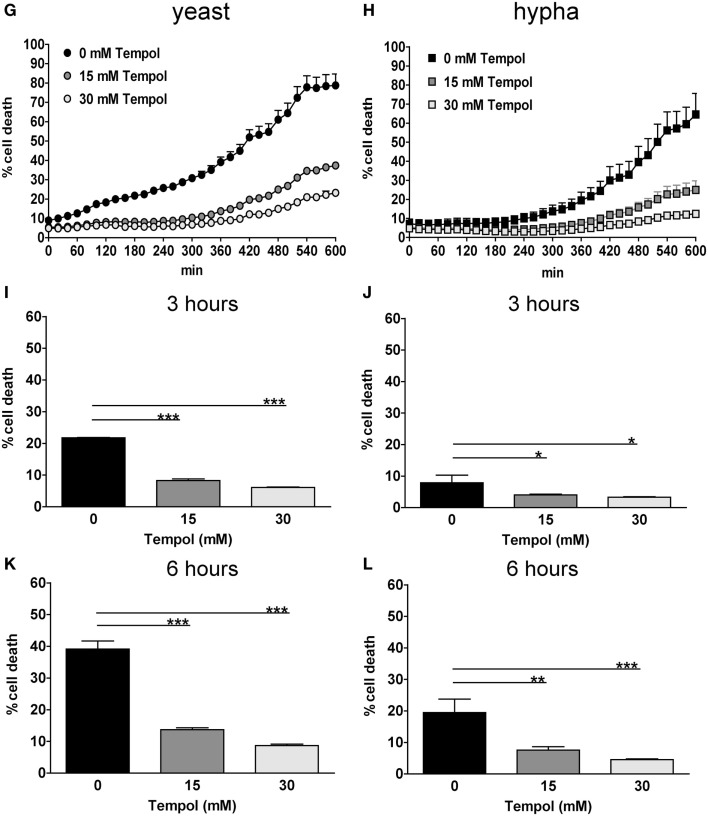
**Tempol prevents cell death induced by different growth forms of *C. albicans*, additional representative results**. The addition of Tempol to the culture medium reduces cell death during infection with *C. albicans*. The rate of cell death of PMNs infected with different growth forms of *C. albicans* was analyzed over a period of 10 h by measurement of DNA-staining by the cell-impermeable dye Sytox Green. The *Y*-axis represents the relative amount of dead cells after normalization to the lysis control (100%). Background values (unstimulated cells) were subtracted from all values. Results are shown for two additional independent donors as rate of PMN cell death induced by yeast **(A,G)** or hyphal cells **(B,H)** of *C. albicans* (MOI 3). Black curves: PMNs infected with *C. albicans*, gray curves: PMNs treated with different concentrations of Tempol. Increasing concentrations of Tempol cause a reduction of PMN cell death upon infection with yeast **(C,I)** or hyphal cells **(D,J)** for 3 h. Percentage of dead cells after 6 h upon infection with yeast or **(E,K)** or hyphal cells of *C. albicans* **(F,L)**. Significance was analyzed by Tukey one-way ANOVA (NS: *P* > 0.05, *P ≤ 0.05, **P ≤ 0.01, and ***P ≤ 0.001). Data are presented as means of three technical replicates ±SD.

We showed that Tempol removed neutrophil-produced ROS effectively in a dose-dependent manner upon PMA and *C. albicans* stimulation. For both stimuli ROS are essential to result in NET formation. In line with this, Tempol reduced the amount of NET release upon PMA and *C. albicans* stimulation. We confirmed that Tempol effectively inhibits NETosis with two independent quantitative assays, using a microscopic approach as well as a fluorescence-based CDA. For PMA-stimulated NET formation both assays correlated extremely well leading to similar results. Using *C. albicans* as a physiological stimulus the microscopic and cell death analyses diverged to a larger extent. Percentage of NET release as determined microscopically was higher up to 6 h after infection with *C. albicans* as compared to neutrophil cell death, as determined by detection of DNA no longer protected by a membrane. Since neutrophil cell death increased up to 50% in later time points (up to 10 h), we assumed that there might be a delay of NET release after decondensation of nuclei. Decondensed nuclei are also classified as NETotic events in the microscopic analysis, whereas nuclei decondense already within an intact plasma membrane and, therefore, are not accessible to the CDA. Additionally, in contrast to the microscopic analysis, the background signal resulting from unstimulated neutrophils was subtracted at any given time point in the CDA. To illustrate the observed effect more clearly we randomly selected two more CDAs showing *C. albicans* infected neutrophils (Figure A2 in Appendix). Overall, neutrophil cell death is lower upon *C. albicans* infection as compared to PMA stimulation, particularly when hyphae are used. The absolute values for neutrophil cell death vary the most for this microbial stimulus, probably due to donor variations and thus we did not combine independently performed CDAs, but rather representative experiments. Usually, neutrophil cell death upon infection with *C. albicans* yeast or hyphae ranges from 25 to 50% after 10 h. Regardless, in each experiment we detected NETs microscopically or by cell death and in both assays Tempol significantly reduced the signal. In contrast to previous findings, we did not observe a significant increase in NET formation upon IL-8 stimulation (Brinkmann et al., 2004; Marcos et al., 2010).

In more recent publications it has been described that alive neutrophils release NETs either from their mitochondria or from the nucleus both through an intact plasma membrane (Yousefi et al., 2009; Yipp et al., 2012). These observations notwithstanding, our findings are still valid for all different types of NET formation that may coexist, as we microscopically validate the presence of NET structures. Moreover, the CDA detects any extracellular DNA that is released irrespective of the fate of the netting neutrophil, since similar membrane-impermeable dyes were used in the mentioned studies (Yousefi et al., 2009; Yipp et al., 2012).

The formation of NETs has been the focus of a large amount of recent studies which addressed the role of these structures in health and disease (Remijsen et al., 2011; Brinkmann and Zychlinsky, 2012). Evidence accumulates that NETs, besides their beneficial role in infection, can also contribute to the pathogenesis of other diseases affecting the immune system in a negative way, e.g., promoting inflammation. The presence of NETs was reported for instance for SLE (Hakkim et al., 2010b; Villanueva et al., 2011; Leffler et al., 2012), autoimmune SVV (Kessenbrock et al., 2009; Nakazawa et al., 2012), allergic asthmatic airways (Dworski et al., 2011), TRALI (Caudrillier et al., 2012; Thomas et al., 2012), amyloidoses (Azevedo et al., 2012), and cancer (Demers et al., 2012).

**Figure A3:**
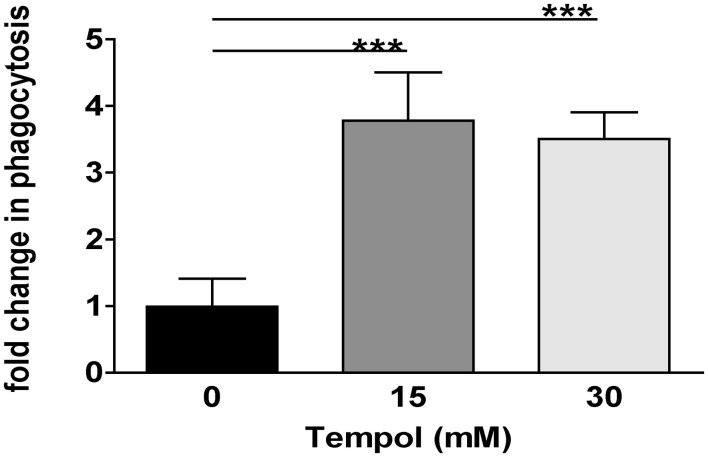
**Tempol enhances PMN phagocytosis of *Staphylococcus aureus* bioparticles**. The effect of Tempol on PMN phagocytosis was analyzed using *Staphylococcus aureus* bioparticles. Cytochalasin D treatment was used as a means to block PMN phagocytosis as a negative control. Black bar: PMN phagocytosis without treatment was set as 1. Gray bars: Fold change of PMN phagocytosis upon treatment with 15 mM Tempol (dark gray) and 30 mM Tempol (light gray) as compared to untreated cells. Significance was analyzed by Tukey one-way ANOVA (***P ≤ 0.001). Data are presented as means from two independent experiments with three technical replicates each ±SD.

The emerging body of evidence for positive as well as negative NET-mediated effects on the health status of the host urge us to search for compounds that are able to interfere with the process of NET formation. A recent study attempted this by systematically screening for compounds that block NET formation. The authors identified NADPH oxidase activation and the Raf-MEK-ERK pathway to be crucial for NET formation (Hakkim et al., 2010a). Since ROS are crucial for many, although certainly not all, NET-inducing stimuli, we reasoned that any ROS interfering compound may used to block NETs. Tempol displays low toxicity in humans, as revealed by a clinical trial where Tempol was applied as topical treatment in a concentration as high as 400 mM (Metz et al., 2004). Systemic administration of 150 μl of 150 mM Tempol in mice resulted in 8.1 mM Tempol in the blood (Davis et al., 2010). Therefore, the amounts required for blockage of NET formation *in vitro* ranging between 10 and 30 mM seem ambitious to be reached *in vivo*, since our *in vitro* findings are 1.2- to 3.7-fold above this *in vivo* concentration. It is very likely that *in vivo* additional factors influence NET formation as compared to *in vitro* experiments. Therefore, future experiments in animals will have to give evidence on whether Tempol reduces NET formation *in vivo*. Application of Tempol seems plausible, since the neutrophils in our assays were not affected by Tempol concentrations as high as 100 mM (Figure A1 in Appendix). Furthermore, Tempol is indeed less toxic than other compounds interfering directly with the activity of the phagocyte oxidase complex, such as DPI (Riganti et al., 2004). Therefore, it might be advantageous to remove ROS after production and not to prevent their production at all. Since Tempol is membrane-permeable, intracellular ROS, which are crucial signaling molecules (Cap et al., 2012), might be removed and this in turn could hamper signaling processes. Of note, we demonstrated that Tempol treatment did not negatively affect phagocytosis of neutrophils, but rather increase engulfment of staphylococcal particles (Figure A3 in Appendix). However, it might not be recommendable to use Tempol in acute infections, since microbicidal mechanisms of phagocytes are undoubtedly affected by removal of ROS.

In conclusion, our study demonstrates that Tempol blocks PMA- and Candida-triggered NET formation *in vitro*. These findings suggest Tempol as a tool to study mechanisms of NET formation and may serve as a promising starting point to investigate the potential of Tempol for the reduction of *in vivo* NET formation.

## Conflict of Interest Statement

The authors declare that the research was conducted in the absence of any commercial or financial relationships that could be construed as a potential conflict of interest.
